# Fish and meat intake in relation to colorectal adenoma in asymptomatic Korean adults

**DOI:** 10.3389/fnut.2024.1432647

**Published:** 2024-09-04

**Authors:** Young Sun Kim, Akinkunmi Paul Okekunle, Sun Young Yang, Ji Hyun Song, Jiyoung Youn, Gabby Yoon Jeong Kwon, Jung Eun Lee

**Affiliations:** ^1^Department of Internal Medicine, Healthcare Research Institute, Seoul National University Hospital Healthcare System Gangnam Center, Seoul, Republic of Korea; ^2^Department of Food and Nutrition, College of Human Ecology, Seoul National University, Seoul, Gwanak-gu, Republic of Korea; ^3^Research Institute of Human Ecology, Seoul National University, Seoul, Gwanak-gu, Republic of Korea; ^4^Department of Biomedical Science, Carnegie Mellon University, Pittsburgh, PA, United States

**Keywords:** fish intake, meat intake, colorectal adenoma, diet, frequency questionnaire

## Abstract

**Introduction:**

Colorectal adenomas are recognized as precursors to colorectal cancer through the adenoma-carcinoma sequence. Identifying modifiable dietary factors that may inhibit cancer progression is critical, but epidemiologic studies in Asian populations are scarce.

**Methods:**

This study explored the impact of fish and meat intake on colorectal adenoma risk among Koreans. The study enrolled asymptomatic adults who visited Seoul National University Hospital Healthcare System Gangnam Center for health check-ups from May to December 2011. All participants underwent screening colonoscopy and completed a validated food frequency questionnaire. The study included 536 adenoma patients, 135 high-risk adenoma patients and 1,122 adenoma-free controls. Using multivariate logistic regression, we calculated odds ratios (ORs) and 95% confidence intervals (CIs) for fish and meat intake related to colorectal adenoma status, significant at *p* < 0.05.

**Results:**

The intake of total fish, meat, red meat, chicken or processed meat showed no clear association with the prevalence of colorectal adenoma after adjusting for age, education, smoking status, alcohol intake, physical activity, body mass index, metabolic syndrome, colorectal cancer family history, total energy intake, and total fruit and vegetable intake. However, higher fish intake was associated with lower odds of high-risk colorectal adenoma, with a significant trend observed across quartiles (P for trend = 0.04). This trend was more pronounced among men than women (P for trend = 0.01).

**Conclusion:**

In conclusion, we observed a significant inverse association between high fish intake and the prevalence of high-risk adenoma, but there were no clear associations between red and processed meat or chicken in the Korean population.

## Introduction

Colorectal cancer is the third most common cause of cancer worldwide (1). The global burden of colorectal cancer is expected to increase to more than 2.2 million new cases and 1.1 million cancer deaths *per annum* by 2030 (2). According to a recently published study (3), the age-standardized incidence rates of colorectal cancer in Korea were 24.4 per 100,000 population (31.7 for men and 17.7 for women). Additionally, the incidence rate of colorectal cancer in the age group of 20 to 49 in Korea was 12.9 per 100,000 population, ranking first among the 42 countries surveyed (4). This rise in colorectal cancer can be attributed to a Westernized dietary lifestyle, increasing population aging, smoking, physical inactivity, and other risk factors. To curb the rising trend, Japan, South Korea, Singapore, and Taiwan have launched nationwide population-based screening programs (5).

Colorectal adenomas are considered precursors to colorectal cancer through the adenoma-carcinoma sequence. Therefore, managing modifiable risk factors such as alcohol, obesity, physical activity, and diet is as important for preventing colorectal cancer as detecting and removing colorectal adenomas through screening tests (6). Current evidence indicates that avoiding processed, charcoal-roasted red meat and increasing vegetable and dietary fiber consumption may have a protective effect against colorectal cancer (1), and the potential role of the intestinal microbiota in mediating the association between diet and colorectal neoplasia has been documented (7).

However, epidemiological studies conducted among Asians on the association between colorectal adenoma, a precancerous stage of colorectal cancer, and dietary habits are limited (7, 8). Despite the westernization of lifestyle and diet, the amount of red meat intake in Korea is still much lower than that of populations in Europe and America (9, 10). According to the results of the recent Korean National Health and Nutrition Examination Surveys, women aged 19–29 years and seniors aged 65 years or older were more likely to consume less than the average requirement for animal protein (11). Additional research is essential to develop a tailored colorectal cancer prevention strategy, particularly involving diverse populations with varied lifestyles, including diet. Therefore, this study investigated the relationship of fish and meat intake with colorectal adenoma.

## Methods

### Study population

The study was approved by the Ethics Review Committee of the Seoul National University Hospital (H-1601-018-731) and was conducted in accordance with the Declaration of Helsinki. Written informed consents were obtained from all participants. The study was retrospective in design, and de-identified data of participants were retrieved from a secured database and medical records for research purposes only in a previously documented cohort (8). Briefly, 1,674 asymptomatic adults who underwent screening colonoscopy at the Seoul National University Hospital Gangnam Healthcare Center in Seoul, Korea (where health examinations for participants were covered either out-of-pocket or by their employers from May to December 2011) were recruited in this study. Participants were excluded from the current analysis for the following reasons: implausible energy intake (energy intake more or less than three standard deviations of the mean log-transformed energy intake; *n* = 5) and history of colorectal cancer (*n* = 11). A total of 1,658 participants were included in the final analysis of this current study (Supplementary Figure S1).

### Assessment of colorectal adenoma

Colorectal adenoma was assessed through colonoscopy by trained gastroenterologists. The level of advancement and anatomic site (proximal colon, distal colon, and rectum) of the polyps were discriminated, and high-risk adenoma was defined as one of the following conditions: villous histology, high-grade dysplasia, or a diameter that was at least 10 mm or at least three adenomas feature in any of the anatomic sites (12). Low-risk adenoma was defined as one or two tubular adenomas <10 mm in diameter with low-grade dysplasia.

### Food and diet assessment

Trained nutritionists administered a validated 106-item frequency questionnaire (FFQ) (13) to evaluate participants’ food and dietary intake during the medical examination before colonoscopic examinations. Details of the FFQ assessment and food consumption estimation have been reported elsewhere (14, 15). Participants provided information on their food consumption frequency and portion sizes in the past 12 months. The food and drink item in the FFQ had nine options ranging from ‘never’ or ‘less than once/month’ to ‘three times/day’ and three portion size options; ‘one-half of a standard serving’, ‘one standard serving’ and ‘one and half of standard serving’. Fish, meat, fruits and vegetable intakes in grams/day were determined by multiplying the frequency of intake by the reported amount. Primary meat sources include red meat, chicken, and processed meat. Total meat included beef, pork, processed meat, organ meat, chicken, and meat soup. Red meat included beef, pork, organ meat and red meat soup. Fish included sashmi, dark meat fish (e.g., mackerel), white meat fish (e.g., flounder), and eel. Also, nutrient intake information for the FFQ was estimated using the food composition table of Korea (16). Fish and meat (including the type of meat sources) in grams per day (g/day) per 1,000 kcal for each participant per day were estimated by dividing fish and/or meat intake in grams by the total caloric intake in a day, multiplied by 1000 (17) and categorized into quartiles.

### Assessment of covariates

Participants provided information on sociodemographics, lifestyle and medical conditions using questionnaires. Also, alcohol intake in grams/day was determined from the FFQ by adding ethanol weight (including the multiplication of quantities and frequencies of types of liquors/alcohol). Physical activity in metabolic-equivalent tasks (MET-mins/week) was estimated based on the average minutes and days spent on moderate, intense or walking (18).

Metabolic syndrome was defined using the harmonized criteria of the International Diabetes Federation, National Heart Lung and Blood Institute, American Heart Association, World Heart Federation, International Atherosclerosis Society, and International Association for the Study of Obesity modified National Cholesterol Education Program Adult Treatment Panel III (NCEP-ATP III) criteria (19). It was defined as the presence of at least three of the following conditions: (a) abdominal obesity, defined as a waist circumference ≥ 90 cm (males) or ≥ 85 cm (females); (b) hypertriglyceridemia, defined as triglycerides ≥ 150 mg/dL, or current use of lipid-lowering medications; (c) high blood pressure, defined as a systolic blood pressure ≥ 130 mmHg or diastolic blood pressure ≥ 85 mmHg, or the use of antihypertensive drugs and (d) diabetes was defined as fasting blood glucose ≥100 mg/dL, or the use of glucose-lowering medications.

### Statistical analysis

Characteristics of participants were presented by colorectal adenoma status (control vs. adenoma group) as n (%) and mean ± standard deviation (SD) for categorical and continuous data, respectively. Polytomous logistic regression models were used to determine the odds ratio (ORs) and 95% CIs for low-risk adenoma and high-risk adenoma by quartiles of fish and meat intakes, adjusting for age (in years, continuous), education (middle school or less, high school, university education and postgraduate), smoking status (never, past, current), alcohol intake (g/day, continuous), physical activity (METS mins/week, continuous), body mass index (kg/m^2^, continuous), metabolic syndrome (no, yes), family history of colorectal cancer (no, yes), total energy intake(kcal/day, continuous), and quartile of total energy-adjusted total fruit and vegetable intake (g/day, quartiles). Furthermore, the median value of the quartile distribution of the fish and meat intakes in g/day per 1,000 kcal was assigned in a continuous model to assess the test for linear trends in the relationship of the fish and meat intakes with colorectal adenoma. The final model was stratified by sex (males, females), smoking status (never or ever), and alcohol use (non-drinkers or current drinkers). For the test for interaction, the likelihood ratio test was used to compare nested models that included cross-product terms with the original models that did not include the term. All statistical analyses were carried out at a two-sided *p*-value *<*0.05 using SAS 9.4 (SAS Institute Inc., Cary, NC, United States).

## Results

### Characteristics of participants by colorectal adenoma status

In all, 536 (32.3%) had colorectal adenoma, with 135 (8.1%) presenting as high-risk colorectal adenoma. The prevalence of high-risk colorectal adenoma was 108 (10.6%) among males but 27 (4.2%) among females. Table 1 outlines in detail the characteristics of participants by colorectal adenoma status. Colorectal adenoma subjects were older and had higher BMI and blood pressure. Similarly, participants with colorectal adenoma presented a higher proportion of current cigarette smoking, higher glucose concentrations with a 183 (34.9%) prevalence of diabetes, and a higher proportion of metabolic syndrome (26.4%) compared to non-cases. A similar observation was recorded when the characteristics were stratified by sex (Supplementary Table S1).

**Table 1:** Characteristics of participants by adenoma status.

	Non-case group	Adenoma group	*p* value
	(*n* = 1,122)	(*n* = 536)	
Age (year), mean ± SD	49.6 ± 8.7	54.1 ± 8.6	<0.0001
Sex, female, n (%)	495 (44.1)	148 (27.6)	<0.0001
Education			
Middle school or less	37 (3.5)	24 (4.7)	0.25
High school	149 (13.8)	81 (15.)	
University Education & Postgraduate	888 (82.7)	406 (79.5)	
Regular cigarette smoking, n (%)			
Never	610 (54.4)	232 (43.3)	0.0001
Past	302 (26.9)	181 (33.8)	
Current	210 (18.7)	123 (22.9)	
Regular alcohol consumption			
Never	305 (27.7)	117 (22.2)	0.05
Past	79 (7.2)	37 (7.0)	
Current	716 (65.1)	373 (70.8)	
Alcohol intake (gram/day)	30.4 ± 39.9	39.2 ± 45.4	0.001
Physical Activity (METS mins/week)	1220.9 ± 2475.1	1348.8 ± 2,531	0.33
Body mass index (kg/m^2^), mean ± SD	23.2 ± 3.0	24.2 ± 2.8	<0.0001
Waist circumference (cm), mean ± SD	83.9 ± 8.2	86.9 ± 7.8	<0.0001
Systolic blood pressure (mmHg), mean ± SD	114.9 ± 13.2	118.6 ± 13.0	<0.0001
Diastolic blood pressure (mmHg), mean ± SD	74.4 ± 10.4	77.1 ± 10.3	<0.0001
High blood pressure, n (%)	363 (32.5)	238 (44.9)	<0.0001
Fasting plasma glucose (mg/dL), mean ± SD	93.1 ± 14.5	98.9 ± 19.8	<0.0001
Glycated hemoglobin (%)	5.6 ± 0.4	5.8 ± 0.7	<0.0001
Diabetes, n (%)	255 (22.9)	183 (34.9)	<0.0001
Total Cholesterol (mg/dL), mean ± SD	199.7 ± 34.4	202.5 ± 35.1	0.13
Triglyceride (mg/dL), mean ± SD	97.5 ± 63.0	112.8 ± 78.4	<0.0001
HDL-cholesterol (mg/dL), mean ± SD	52.9 ± 11.2	50.8 ± 10.8	0.0003
LDL-cholesterol (mg/dL), mean ± SD	125.3 ± 30.8	128.4 ± 29.7	0.05
Metabolic syndrome, n (%)	183 (16.3)	140 (26.4)	<0.0001
Colorectal cancer family history, n (%)	61 (17.8)	37 (25.5)	0.05
Total energy intake (kcal/day), mean ± SD	1866.2 ± 535.5	1880.2 ± 545.7	0.62

SD, standard deviation; HDL, high-density lipoprotein-cholesterol; LDL, low-density lipoprotein-cholesterol; MET, the metabolic equivalent of task. High blood pressure was defined as systolic blood pressure ≥ 130 mmHg or diastolic blood pressure ≥ 85 mmHg or the use of antihypertensive drug treatment or a history of hypertension. Diabetes was defined as fasting blood glucose ≥ 100 mg/dL or the use of glucose-lowering medications. Metabolic syndrome was defined as at least three of any of the following conditions; elevated waist circumference (≥90 cm – males ≥ 85 cm - females), elevated triglycerides (≥150 mg/dL or drug treatment for elevated triglycerides), reduced HDL-c (<40 mg/dL—males; <50 mg/dL—females), high blood pressure or diabetes.

### Association of fish and meat intake with colorectal adenoma

Total fish and meat intakes in g/d per 1,000 kcal by quartiles of fish and meat intakes was 18.2 (12.1, 23.0)—first quartile, 33.9 (30.7, 37.5)—second quartile, 49.5 (45.4, 54.3)—third quartile and 74.7 (66.1, 91.6)—fourth quartile (Table 2).

**Table 2:** Polytomous regression models for the association of meat and fish intake with risk of adenoma (All participants).^1^

	Median (IQR) intake (g/day per 1,000 kcal)	Median (IQR) intake (g/day per)	*N*	Low risk adenoma group (*n* = 401)		High risk adenoma group (*n* = 135)	
				*Cases, (n)*	OR (95%CI)^1^	*Cases, (n)*	OR (95%CI)^1^
Meat and fish							
Q1	18.2 (12.1, 23.0)	29.8 (18.8, 40.7)	414	99	1.00	40	1.00
Q2	33.9 (30.7, 37.5)	60.5 (50.0, 73.9)	415	104	1.07 (0.77, 1.50)	34	0.92 (0.55, 1.55)
Q3	49.5 (45.4, 54.3)	90.2 (74.1, 110.2)	415	98	1.01 (0.72, 1.42)	28	0.77 (0.44, 1.34)
Q4	74.7 (66.1, 91.6)	149.5 (113.6, 192.9)	414	100	1.08 (0.77, 1.52)	33	1.00 (0.58, 1.72)
*P*-trend					0.73		0.94
Meats only							
Q1	9.7 (5.2, 13.0)	15.8 (8.3, 22.0)	414	101	1.00	39	1.00
Q2	21.9 (18.5, 24.7)	39.2 (31.4, 49.4)	415	103	1.05 (0.75, 1.47)	41	1.08 (0.65, 1.80)
Q3	34.1 (30.6, 37.6)	60.4 (49.2, 76.2)	415	95	1.00 (0.71, 1.40)	21	0.64 (0.35, 1.16)
Q4	56.2 (48.7, 70.0)	112.2 (86.3, 148.6)	414	102	1.17 (0.83, 1.66)	34	1.12 (0.65, 1.93)
*P*-trend					0.40		0.92
Red meat only							
Q1	8.3 (4.2, 11.3)	13.3 (6.7, 19.2)	414	102	1.00	35	1.00
Q2	19.2 (16.1, 21.9)	33.8 (27.1, 42.2)	415	98	0.99 (0.70, 1.38)	42	1.32 (0.78, 2.21)
Q3	30.4 (27.5, 33.8)	54.2 (44.0, 68.3)	415	97	0.99 (0.70, 1.39)	25	0.79 (0.44, 1.42)
Q4	51.3 (43.6, 64.7)	103.1 (77.6, 135.0)	414	104	1.17 (0.83, 1.65)	33	1.23 (0.70, 2.16)
*P*-trend					0.33		0.76
Poultry meat only							
Q1	0.0 (0.0, 0.5)	0.0 (0.0, 1.3)	414	101	1.00	43	1.00
Q2	1.2 (1.0, 1.4)	2.5 (1.3, 2.5)	415	126	1.50 (1.08, 2.08)	42	1.28 (0.77, 2.10)
Q3	2.3 (1.9, 2.8)	3.4 (2.5, 6.3)	415	82	0.88 (0.62, 1.24)	29	0.92 (0.53, 1.59)
Q4	4.4 (3.8, 6.0)	8.5 (6.3, 16.1)	414	92	1.02 (0.72, 1.45)	21	0.72 (0.39, 1.30)
*P*-trend					0.43		0.16
Processed meat only							
T1	0.0 (0.0, 0.0)	0.0 (0.0, 0.0)	878	220	1.00	84	1.00
T2	0.5 (0.4, 0.7)	0.7 (0.7, 1.3)	366	90	1.09 (0.80, 1.48)	25	0.94 (0.56, 1.56)
T3	2.0 (1.4, 3.4)	3.3 (3.3, 8.6)	414	91	1.13 (0.82, 1.54)	26	1.16 (0.69, 1.95)
*P*-trend					0.49		0.57
Fish only							
Q1	3.6 (1.7, 4.9)	6.3 (3.2, 9.0)	414	86	1.00	37	1.00
Q2	8.3 (7.1, 9.4)	15.2 (12.0, 18.6)	415	102	1.11 (0.79, 1.57)	30	0.75 (0.44, 1.30)
Q3	13.5 (11.8, 15.1)	24.6 (19.0, 30.8)	415	109	1.27 (0.90, 1.79)	45	1.23 (0.73, 2.06)
Q4	24.9 (20.3, 32.6)	45.4 (34.4, 63.8)	414	104	0.99 (0.70, 1.40)	23	0.51 (0.28, 0.92)
*P*-trend					0.77		0.04

IQR, interquartile range; OR, odds ratio; CI, confidence interval; Q, quartile; T, tertile.

1 Model was adjusted for age (in years, continuous), education (middle school or less, high school, university graduate and postgraduate), smoking status (never, past, current), alcohol intake (g/day, continuous), physical activity (METS mins/week, continuous), body mass index (kg/m^2^, continuous), metabolic syndrome (no, yes), colorectal cancer family history (no, yes), total energy intake(kcal/day, continuous), and total fruit and vegetable intake (g/day per 1,000 kcal, quartiles).

In the polytomous logistic regression models, total fish and meat intakes were unrelated with both low-risk colorectal adenoma (*P* for trend = 0.73) and high-risk colorectal adenoma (*P* for trend = 0.94) after adjusting for age, education, smoking status, alcohol intake, physical activity, body mass index, metabolic syndrome, colorectal cancer family history, total energy intake, and total fruit and vegetable intake. No clear observations were observed for total meat, red meat, chicken, and processed meats. However, higher fish intakes were associated with a low odd of high-risk colorectal adenoma. Using the first quartile as a reference, the ORs (95% CIs) were 0.75 (0.44, 1.30) in the second quartile, 1.23 (0.73, 2.06) in the third quartile and 0.51 (0.28, 0.92) in the fourth quartile (*P* for trend = 0.04). The median (IQR) of fish intakes in g/d per 1,000 kcal by quartiles of fish intakes were 3.6 (1.7, 4.9) in the first quartile, 8.3 (7.1, 9.4) in the second quartile, 13.5 (11.8, 15.1) in the third quartile and 24.9 (20.3, 32.6) in the fourth quartile. The inverse association remained after additional adjustment for red meat intakes (*P* for trend = 0.03; Supplementary Table S2). Similarly, the statistically inverse association between fish consumption and colorectal adenoma remained unchanged when we additionally adjusted for seaweed consumption (given its rampant consumption among Koreans).

When the associations were stratified by sex (Table 3), higher fish intakes were associated with a low odd of high-risk colorectal adenoma among males; ORs (95% CIs), 0.63 (0.34, 1.14) in the second quartile, 0.80 (0.44, 1.45) in the third quartile and 0.36 (0.18, 0.71) in the fourth quartile (*P* for trend = 0.01), but not among females ORs (95% CIs), 1.57 (0.42, 5.88) in the second quartile, 2.18 (0.66, 7.17) in third quartile and 0.94 (0.24, 0.3.73) in fourth quartile (*P* for trend = 0.78). The association was stronger among males with additional adjustment for red meat intakes (*P* for trend = 0.003), and there was no evidence of interaction (*P* for interaction = 0.16; Supplementary Table S2). Increased consumption of meat, or red meat, was associated with a higher incidence of low-risk colorectal adenomas in males (*P* for trend = 0.04). In contrast, a reduced intake of meat and fish correlated with an increased occurrence of low-risk colorectal adenomas among females (*P* for trend = 0.05; Table 3). However, there was no statistically significant association between intake of meat types and colorectal adenoma when the low-risk and high-risk adenoma groups were combined as a single outcome in logistic regression models (Supplementary Table S3).

**Table 3:** Polytomous regression models for the association of meat and fish intake with odds of adenoma stratified by sex (males vs. females).^1^

	Males						Females					
	Median (IQR) intake (g/day per 1,000 kcal)	*N*	Low risk adenoma group (*n* = 280)		High risk adenoma group (*n* = 108)		Median (IQR) intake (g/day per 1,000 kcal)	*N*	Low risk denoma group (*n* = 121)		High riskadenoma group (*n* = 27)	
			*Cases, (n)*	OR (95%CI)^1^	*Cases, (n)*	OR (95%CI)^1^			*Cases, (n)*	OR (95%CI)^1^	*Cases, (n)*	OR (95%CI)^1^
Meat and fish												
Q1	20.3 (14.8, 25.1)	253	58	1.00	36	1.00	14.7 (8.7, 19.1)	160	38	1.00	9	1.00
Q2	35.4 (31.6, 38.8)	254	75	1.40 (0.92, 2.14)	24	0.71 (0.39, 1.28)	31.7 (27.5, 34.8)	161	34	0.90 (0.51, 1.57)	6	0.86 (0.27, 2.74)
Q3	51.1 (46.5, 55.5)	254	73	1.36 (0.89, 2.09)	22	0.68 (0.37, 1.26)	48.0 (43.4, 51.8)	161	27	0.67 (0.37, 1.20)	4	0.46 (0.12, 1.74)
Q4	75.9 (68.0, 89.6)	254	74	1.51 (0.98, 2.32)	26	0.90 (0.49, 1.64)	72.9 (63.0, 91.6)	161	22	0.57 (0.31, 1.05)	8	1.59 (0.50, 5.09)
*P*-trend				0.11		0.75				0.05		0.60
Meats only												
Q1	12.2 (7.5, 15.0)	253	65	1.00	34	1.00	7.0 (3.0, 10.2)	160	41	1.00	7	1.00
Q2	23.6 (20.8, 26.4)	254	68	1.16 (0.76, 1.77)	31	1.01 (0.57, 1.78)	17.8 (15.2, 21.9)	161	32	0.75 (0.42, 1.31)	10	1.72 (0.57, 5.15)
Q3	35.4 (32.6, 39.0)	254	72	1.33 (0.87, 2.03)	17	0.68 (0.35, 1.32)	31.7 (28.5, 35.1)	161	25	0.59 (0.32, 1.08)	3	0.62 (0.14, 2.85)
Q4	56.4 (50.1, 70.3)	254	75	1.55 (1.01, 2.39)	26	1.16 (0.63, 2.13)	55.1 (46.0, 69.4)	161	23	0.56 (0.30, 1.03)	7	1.90 (0.55, 6.61)
*P*-trend				0.04		0.78				0.06		0.51
Red meat only												
Q1	10.8 (6.4, 13.0)	253	65	1.00	34	1.00	5.8 (2.0, 8.3)	160	40	1.00	7	1.00
Q2	20.9 (18.6, 23.6)	254	66	1.12 (0.73, 1.70)	28	0.95 (0.53, 1.70)	15.7 (13.0, 18.6)	161	30	0.74 (0.42, 1.31)	10	1.49 (0.50, 4.44)
Q3	32.0 (28.9, 35.1)	254	75	1.38 (0.91, 2.10)	20	0.78 (0.41, 1.47)	27.9 (24.5, 30.7)	161	25	0.60 (0.33, 1.10)	2	0.33 (0.06, 1.82)
Q4	52.6 (44.7, 65.8)	254	74	1.48 (0.97, 2.27)	26	1.12 (0.61, 2.06)	48.2 (41.1, 64.5)	161	26	0.67 (0.37, 1.22)	8	2.05 (0.62, 6.80)
*P*-trend				0.05		0.77				0.20		0.41
Poultry meat only												
Q1	0.0 (0.0, 0.6)	253	70	1.00	31	1.00	0.0 (0.0, 0.0)	160	30	1.00	12	1.00
Q2	1.3 (1.1, 1.5)	254	82	1.51 (1.00, 2.27)	37	1.61 (0.91, 2.86)	1.1 (0.9, 1.4)	161	43	1.74 (0.99, 3.07)	5	0.55 (0.17, 1.77)
Q3	2.4 (2.0, 2.9)	254	60	0.92 (0.60, 1.40)	21	0.85 (0.45, 1.61)	2.1 (1.8, 2.5)	161	24	0.91 (0.49, 1.69)	8	1.00 (0.35, 2.84)
Q4	4.4 (3.8, 6.0)	254	68	1.12 (0.73, 1.71)	19	0.95 (0.49, 1.85)	4.4 (3.8, 5.9)	161	24	0.99 (0.52, 1.87)	2	0.22 (0.04, 1.27)
*P*-trend				0.84		0.47				0.47		0.14
Processed meat only												
T1	0.0 (0.0, 0.0)	505	143	1.00	64	1.00	0.0 (0.0, 0.0)	373	77	1.00	20	1.00
T2	0.3 (0.4, 0.5)	172	44	1.00 (0.66, 1.52)	17	1.10 (0.59, 2.04)	0.5 (0.4, 0.6)	109	22	1.06 (0.60, 1.90)	2	0.56 (0.11, 2.87)
T3	1.6 (1.0, 2.6)	338	93	1.28 (0.90, 1.83)	27	1.10 (0.64, 1.89)	2.1 (1.4, 3.4)	161	22	0.76 (0.42, 1.37)	5	1.52 (0.46, 5.00)
*P*-trend				0.15		0.74				0.35		0.47
Fish only												
Q1	4.2 (2.4, 5.4)	253	58	1.00	33	1.00	2.7 (1.1, 4.1)	160	27	1.00	6	1.00
Q2	8.8 (7.8, 9.8)	254	69	1.09 (0.71, 1.67)	26	0.63 (0.34, 1.14)	7.1 (6.2, 8.6)	161	38	1.64 (0.90, 2.99)	6	1.57 (0.42, 5.88)
Q3	13.6 (12.0, 15.3)	254	75	1.25 (0.82, 1.92)	31	0.80 (0.44, 1.45)	13.0 (11.1, 14.7)	161	30	1.12 (0.60, 2.07)	10	2.18 (0.66, 7.17)
Q4	24.2 (19.7, 32.0)	254	78	1.05 (0.68, 1.62)	18	0.36 (0.18, 0.71)	25.7 (21.2, 33.6)	161	26	0.95 (0.50, 1.79)	5	0.94 (0.24, 0.3.73)
*P*-trend				0.91		0.01				0.35		0.78

IQR, interquartile range; OR, odds ratio; CI, confidence interval; Q, quartile; T, tertile.

1 Model was adjusted for age (in years, continuous), education (middle school or less, high school, university graduate and postgraduate), smoking status (never, past, current), alcohol intake (g/day, continuous), physical activity (METS mins/week, continuous), body mass index (kg/m^2^, continuous), metabolic syndrome (no, yes), colorectal cancer family history (no, yes), total energy intake (kcal/day, continuous), and total fruit and vegetable intake (g/day per 1,000 kcal, quartiles).

Subsequent stratification based on smoking status and alcohol consumption likewise revealed no significant relationship between intake of meat type and adenoma risk (Tables 4, 5). The association remained null after further adjustment for red meat intake (Supplementary Table S4).

**Table 4:** Polytomous regression models for the association of meat and fish intake with odds of adenoma stratified by smoking status (never vs. ever).^1^

	Never smokers only						Ever smokers only					
	Median (IQR) intake (g/day per 1,000 kcal)	*N*	Low risk adenoma group (*n* = 186)		High risk adenoma group (*n* = 46)		Median (IQR) intake (g/day per 1,000 kcal)	*N*	Low risk adenoma group (*n* = 215)		High risk adenoma group (*n* = 89)	
			*Cases, (n)*	OR (95%CI)^1^	*Cases, (n)*	OR (95%CI)^1^			*Cases, (n)*	OR (95%CI)^1^	*Cases, (n)*	OR (95%CI)^1^
Meat and fish												
Q1	16.7 (11.0, 21.9)	238	58	1.00	19	1.00	19.0 (12.8, 23.7)	176	41	1.00	21	1.00
Q2	33.6 (30.9, 37.3)	200	40	0.76 (0.47, 1.22)	10	0.67 (0.29, 1.55)	33.9 (30.5, 37.6)	215	64	1.55 (0.96, 2.51)	24	1.09 (0.55, 2.15)
Q3	49.2 (45.1, 54.1)	108	46	0.95 (0.60, 1.52)	9	0.64 (0.26, 1.55)	49.9 (45.9, 54.4)	207	52	1.16 (0.70, 1.91)	19	0.87 (0.43, 1.78)
Q4	75.5 (66.7, 94.1)	196	42	0.91 (0.56, 1.47)	8	0.70 (0.28, 1.77)	73.7 (66.0, 88.0)	218	58	1.32 (0.81, 2.17)	25	1.21 (0.61, 2.41)
*P*-trend				0.89		0.41				0.61		0.68
Meats only												
Q1	9.5 (5.2, 12.9)	258	62	1.00	19	1.00	10.1 (5.1, 13.1)	156	39	1.00	20	1.00
Q2	21.8 (18.2, 24.9)	197	44	0.99 (0.62, 1.58)	14	1.06 (0.48, 2.32)	21.9 (19.0, 24.5)	218	59	1.17 (0.71, 1.93)	27	1.06 (0.54, 2.09)
Q3	34.3 (30.8, 38.6)	196	36	0.85 (0.52, 1.39)	5	0.43 (0.15, 1.27)	34.1 (30.5, 37.0)	219	59	1.21 (0.74, 1.99)	16	0.73 (0.35, 1.53)
Q4	57.9 (49.3, 76.8)	191	44	1.14 (0.7, 1.86)	8	0.8 (0.31, 2.07)	55.1 (48.5, 67.5)	223	58	1.24 (0.74, 2.06)	26	1.30 (0.65, 2.06)
*P*-trend				0.67		0.43				0.48		0.53
Red meat only												
Q1	8.2 (4.3, 11)	262	62	1.00	16	1.00	8.8 (4.1, 11.6)	152	40	1.00	19	1.00
Q2	19.4 (16.1, 22.1)	202	44	1.01 (0.63, 1.60)	17	1.63 (0.75, 3.56)	19.1 (16.2, 21.5)	213	54	0.99 (0.60, 1.64)	25	1.05 (0.52, 2.10)
Q3	30.4 (27.6, 33.2)	184	32	0.84 (0.51, 1.40)	6	0.72 (0.26, 2.05)	30.5 (27.3, 33.9)	231	65	1.15 (0.70, 1.87)	19	0.76 (0.37, 1.56)
Q4	53 (43.2, 68.1)	194	48	1.26 (0.78, 2.02)	7	0.84 (0.31, 2.26)	51.1 (43.9, 60.5)	220	56	1.09 (0.66, 1.82)	26	1.34 (0.66, 2.71)
*P*-trend				0.37		0.51				0.63		0.44
Poultry meat only												
Q1	0.0 (0.0, 0.6)	229	55	1.00	21	1.00	0.0 (0.0, 0.5)	185	46	1.00	22	1.00
Q2	1.2 (1.0, 1.4)	192	50	1.19 (0.74, 1.90)	10	0.70 (0.30, 1.61)	1.2 (1.0, 1.5)	223	76	1.88 (1.18, 2.99)	32	1.89 (0.99, 3.62)
Q3	2.2 (1.9, 2.7)	221	39	0.86 (0.53, 1.41)	13	0.91 (0.41, 2.01)	2.4 (2.0, 2.9)	194	43	0.97 (0.58, 1.61)	16	0.93 (0.44, 1.96)
Q4	4.4 (3.8, 5.7)	200	42	1.06 (0.65, 1.74)	2	0.17 (0.04, 0.78)	4.4 (3.8, 6.2)	214	50	1.05 (0.64, 1.72)	19	1.15 (0.56, 2.36)
*P*-trend				0.97		0.03				0.36		0.73
Processed meat only												
T1	0.0 (0.0, 0.0)	471	110	1.00	30	1.00	0.0 (0.0, 0.0)	407	110	1.00	54	1.00
T2	0.5 (0.4, 0.7)	172	38	1.12 (0.71, 1.79)	7	0.95 (0.37, 2.41)	0.5 (0.4, 0.7)	194	52	1.07 (0.71, 1.61)	18	0.90 (0.49, 1.65)
T3	2.2 (1.4, 3.7)	199	38	1.16 (0.72, 1.87)	9	1.39 (0.59, 3.38)	1.9 (1.3, 3.3)	215	53	1.09 (0.71, 1.67)	17	1.02 (0.54, 1.93)
*P*-trend				0.58		0.46				0.71		0.95
Fish only												
Q1	3.4 (1.8, 4.8)	228	45	1.00	17	1.00	3.8 (1.7, 5.1)	186	41	1.00	20	1.00
Q2	8.2 (7.0, 9.4)	200	48	1.18 (0.72, 1.93)	7	0.49 (0.18, 1.31)	8.4 (7.3, 9.5)	215	54	1.09 (0.67, 1.77)	23	0.80 (0.40, 1.60)
Q3	13.1 (11.6, 14.8)	198	47	1.13 (0.69, 1.85)	15	1.13 (0.50, 2.55)	13.6 (12, 15.3)	217	62	1.42 (0.87, 2.30)	30	1.12 (0.57, 2.20)
Q4	24.9 (20.3, 32.9)	216	46	0.87 (0.53, 1.44)	7	0.40 (0.15, 0.1.07)	24.9 (20.4, 32)	198	58	1.14 (0.69, 1.88)	16	0.51 (0.24, 0.1.11)
*P*-trend				0.40		0.13				0.64		0.11

IQR, interquartile range; OR, odds ratio; CI, confidence interval; Q, quartile; T, tertile.

1 Model was adjusted for age (in years, continuous), education (middle school or less, high school, university graduate and postgraduate), alcohol intake (g/day, continuous), physical activity (METS mins/week, continuous), body mass index (kg/m^2^, continuous), metabolic syndrome (no, yes), colorectal cancer family history (no, yes), total energy intake (kcal/day, continuous), and total fruit and vegetable intake (g/day per 1,000 kcal, quartiles).

**Table 5:** Polytomous logistic regression models for the association of meat and fish intake with risk of adenoma stratified by current alcohol drinking (non-drinkers vs. current drinkers).^1^

	Non-drinkers						Current drinkers					
	Median (IQR) intake (g/day per 1,000 kcal)	*N*	Low risk adenoma group (*n* = 120)		High risk adenoma group (*n* = 34)		Median (IQR) intake (g/day per 1,000 kcal)	*N*	Low risk adenoma group (*n* = 273)		High risk adenoma group (*n* = 100)	
			*Cases, (n)*	OR (95%CI)^1^	*Cases, (n)*	OR (95%CI)^1^			*Cases, (n)*	OR (95%CI)^1^	*Cases, (n)*	OR (95%CI)^1^
Meat and fish												
Q1	16.4 (9.6, 21.4)	167	41	1.00	12	1.00	19.0 (14.1, 23.6)	237	55	1.00	28	1.00
Q2	33.1 (30.2, 36.7)	121	29	0.93 (0.52, 1.66)	5	0.43 (0.13, 1.45)	34.0 (30.8, 37.6)	288	73	1.15 (0.75, 1.75)	29	0.97 (0.53, 1.77)
Q3	49.1 (45.4, 54.4)	129	29	0.92 (0.52, 1.64)	7	0.66 (0.22, 1.94)	49.5 (45.4, 54.2)	280	67	1.04 (0.68, 1.60)	21	0.73 (0.38, 1.41)
Q4	74.8 (64.9, 92.5)	121	21	0.64 (0.34, 1.20)	10	0.87 (0.31, 2.44)	74.0 (66.6, 89.3)	284	78	1.34 (0.87, 2.05)	22	0.94 (0.49, 1.81)
*P*-trend				0.19		0.98				0.23		0.72
Meats only												
Q1	8.9 (4.5, 12.9)	189	44	1.00	9	1.00	10.5 (6.5, 13.1)	214	55	1.00	30	1.00
Q2	21.4 (18.3, 24.1)	118	31	1.35 (0.76, 2.39)	11	2.10 (0.74, 5.97)	22.2 (18.6, 24.8)	291	69	0.88 (0.57, 1.36)	30	0.71 (0.39, 1.30)
Q3	34.2 (31.1, 38.9)	117	20	0.72 (0.38, 1.35)	4	0.52 (0.13, 2.07)	34.1 (30.4, 37.4)	294	74	1.03 (0.67, 1.58)	17	0.54 (0.28, 1.07)
Q4	57.2 (50.7, 75.6)	114	25	1.04 (0.56, 1.92)	10	1.82 (0.61, 5.49)	55.8 (48.1, 68.0)	290	75	1.14 (0.73, 1.78)	23	0.79 (0.41, 1.50)
*P*-trend				0.76		0.53				0.33		0.52
Red meat only												
Q1	7.2 (3.6, 10.7)	184	43	1.00	9	1.00	9.0 (5.1, 11.7)	219	57	1.00	26	1.00
Q2	18.2 (15.8, 21.5)	124	30	1.18 (0.67, 2.11)	11	1.94 (0.68, 5.51)	19.5 (16.2, 21.9)	284	64	0.82 (0.53, 1.27)	31	0.97 (0.52, 1.78)
Q3	30.3 (27.6, 33.3)	111	21	0.82 (0.44, 1.54)	5	0.66 (0.18, 2.43)	30.5 (27.4, 33.9)	301	76	0.98 (0.64, 1.50)	20	0.67 (0.34, 1.31)
Q4	51.2 (43.1, 66.9)	119	26	0.99 (0.54, 1.80)	9	1.39 (0.46, 4.25)	51.3 (43.8, 64.1)	285	76	1.16 (0.76, 1.81)	23	1.00 (0.51, 1.94)
*P*-trend				0.77		0.87				0.22		0.89
Poultry meat only												
Q1	0.0 (0.0, 0.6)	161	40	1.00	15	1.00	0.0 (0.0, 0.5)	244	59	1.00	28	1.00
Q2	1.1 (1.0, 1.4)	125	36	1.27 (0.72 2.22)	8	0.74 (0.27, 2.01)	1.2 (1.0, 1.5)	284	86	1.50 (1.00, 2.27)	33	1.44 (0.79, 2.61)
Q3	2.2 (1.9, 2.6)	128	19	0.64 (0.34, 1.21)	7	0.79 (0.27, 2.27)	2.4 (1.9, 2.8)	280	63	1.03 (0.67, 1.60)	22	0.97 (0.50, 1.87)
Q4	4.3 (3.8, 6.1)	124	25	0.89 (0.48, 1.65)	4	0.43 (0.12, 1.55)	4.5 (3.8, 5.7)	281	65	1.06 (0.69, 1.64)	17	0.83 (0.41, 1.66)
*P*-trend				0.43		0.22				0.66		0.34
Processed meat only												
T1	0.0 (0.0, 0.0)	330	79	1.00	25	1.00	0.0 (0.0, 0.0)	531	136	1.00	58	1.00
T2	0.5 (0.4, 0.7)	106	21	0.84 (0.47, 1.50)	4	0.59 (0.18, 1.91)	0.5 (0.4, 0.7)	253	67	1.23 (0.85, 1.77)	21	1.12 (0.63, 1.99)
T3	2.0 (1.4, 3.0)	102	20	1.00 (0.54, 1.86)	5	1.02 (0.32, 3.31)	2.1 (1.4, 3.5)	305	70	1.19 (0.82, 1.74)	21	1.29 (0.71, 2.33)
*P*-trend				0.98		0.98				0.44		0.43
Fish only												
Q1	3.4 (1.7, 4.8)	151	32	1.00	10	1.00	3.7 (1.8, 4.9)	255	50	1.00	27	1.00
Q2	8.6 (7.2, 9.5)	127	34	1.31 (0.72, 2.38)	5	0.65 (0.19, 2.23)	8.2 (7.1, 9.3)	281	65	1.09 (0.71, 1.67)	25	0.77 (0.41, 1.44)
Q3	13.5 (11.5, 15.2)	123	30	1.15 (0.63, 2.12)	14	1.88 (0.70, 5.11)	13.5 (11.9, 15.1)	288	79	1.37 (0.89, 2.10)	30	0.96 (0.52, 1.79)
Q4	27.1 (22.1, 34.2)	137	24	0.65 (0.35, 1.23)	5	0.46 (0.14, 1.57)	24.0 (19.8, 31.8)	265	79	1.29 (0.84, 2.00)	18	0.54 (0.27, 1.09)
*P*-trend				0.08		0.28				0.22		0.12

IQR, interquartile range; OR, odds ratio; CI, confidence interval; Q, quartile; T, tertile.

1 Model was adjusted for age (in years, continuous), education (middle school or less, high school, university graduate and postgraduate), smoking status (never, past, current), physical activity (METS mins/week, continuous), body mass index (kg/m^2^, continuous), metabolic syndrome (no, yes), colorectal cancer family history (no, yes), total energy intake (kcal/day, continuous), and total fruit and vegetable intake (g/day per 1,000 kcal, quartiles).

## Discussion

This study investigated the relationship between the consumption of fish and meat and the odds of colorectal adenoma. The present study’s findings indicate that a higher fish intake was significantly associated with a decreased chance of high-risk colorectal adenoma but not low-risk colorectal adenoma. This inverse association remained after considering the consumption of red meat. Nonetheless, no clear relationship was discovered between meat consumption (red meat, chicken, or processed meat) and the odds of developing low or high-risk colorectal adenoma. As food material and metabolites come into direct contact with the colonic mucosa, it is reasonable to assume that dietary patterns can impact the risk of developing colorectal cancer (20).

Fish is a major source of omega-3 fatty acids, which have been shown to have anti-inflammatory and anticarcinogenic effects that may help protect against colorectal cancer (21). These fatty acids regulate the production of proinflammatory prostaglandins and hydroxyeicosatetraenoic acid through the cyclooxygenase and lipoxygenase pathways, which are known to play significant roles in inflammation, cell proliferation and angiogenesis—all key factors in cancer progression (21). In addition, omega-3 polyunsaturated fatty acids have been associated with a higher intestinal microbial diversity, which improves host immune function and may eventually decrease the risk of developing colorectal cancer (22). On the other hand, the association between fish consumption and colorectal cancer risk may be due to a substitution effect, as people who eat more fish are likely to generally eat less red meat with healthier lifestyles than those who prefer red meat (23). According to a systematic review of 22 prospective cohorts and 19 case–control studies, fish consumption can reduce the risk of colorectal cancer by 12% (24). Another meta-analysis of 20 prospective cohort studies also found that fish consumption was associated with a reduced risk of colorectal cancer (RR = 0.93; 95%CI: 0.87–0.99; *p* < 0.01) (21). A recent study found that the protective effect of canned fish on colorectal cancer substantially equalled that of fresh fish, and the consumption of both types of fish provided an even more significant effect, suggesting that fish consumption offers protection from colorectal cancer; however, even though processed (25). Our study included the results of fresh cooked fish and raw fish. We did not consider fermented fish in our study.

However, the results of studies on colorectal adenoma, a pre-stage of colorectal cancer, and dietary risk factors are still controversial (26). Furthermore, most of the included studies were performed in Europe and United States. A meta-analysis of 21 independent observational studies (16 case–control and five cohort studies) from 23 publications showed no association between fish intake and risk of colorectal adenoma (26). In the present study, higher fish intakes were significantly associated with a low odd of high-risk colorectal adenoma but not low-risk colorectal adenoma. These results suggest that fish intake in the long term should significantly affect the late stage of the adenoma colorectal cancer sequence and manifestation. There is a substantial lag between exposure time to a risk factor (or protective factor) and the incidence of cancer (27). For colorectal cancer, the time from the formation of adenoma to colorectal cancer occurrence takes about 10–15 years, or even more (28).

Sex- and gender-associated distinctions in colorectal cancer development exist (29, 30). However, a recent review article highlights that only half of the studies provided sex-specific risk estimates despite potential variances in colorectal cancer risk associated with sex-related differences in dietary factors (29). In the present study, when the associations were stratified by sex, higher fish intakes were associated with a low odd of high-risk colorectal adenoma among males. It may be challenging to explain perfectly the gender differences in colorectal adenoma risk, but it may be related to the differences in diet patterns or sex hormones among men and women. Individual risk factors for colorectal adenoma, as well as individual components of diet, may have different contributions to colorectal adenoma by sex. For example, given that sociological and cultural aspect of alcohol drinking vary by sex, and that the toxic threshold of ethanol may differ by sex, it may be important to provide specific summaries for women and men (29, 30). Estrogen exposure may be linked to insulin-mediated growth regulation pathways and thus could have affected colorectal carcinogenesis (29, 30). Experimental studies in female mice with high estrogenic backgrounds show increased estrogen receptor-α expression, reduced apoptosis, and inflammation markers in the colonic mucosa with high phytoestrogen intake (31). Large population-based cohort studies need to report sex-specific estimates of dietary risk factors to provide better guidelines for cancer-preventive dietary intake.

In the present study, the intake of total meat, red meat, chicken or processed meat showed no clear association with colorectal adenoma. A diet rich in red and processed meat is associated with colorectal carcinogenesis, attributed to compounds that damage the intestinal mucosa and promote cancer development (32). Mechanisms include forming carcinogenic substances like heterocyclic amines and polycyclic aromatic hydrocarbons during meat cooking (33). The link between heme iron in red meat and colorectal cancer involves pro-oxidative properties inducing the oxidation of dietary polyunsaturated fatty acids (34). This oxidation results in the production of cytotoxic and genotoxic substances, such as malondialdehyde or 4-hydroxynonenal, contributing to colorectal cancer development (34). Epidemiological studies have demonstrated that long-term consumption of red and processed meat is associated with an increased risk of colon cancer (35, 36). In contrast, other studies have shown no correlation between meat consumption and colorectal cancer risk (37, 38). A pooled analysis of six large-scale population-based cohort studies performed in Japan (39) showed that higher intake of total red meat was not significantly associated with an increased risk of colorectal cancer and its subsites in both men and women. Studies on the possible association between white meat (such as poultry—chicken, turkey, duck, and goose—and rabbit) or eggs and colorectal cancer risk are limited, and the results are unclear (40). Explaining discrepancies with prior studies poses challenges due to variations in study design, region, case numbers, FFQ type, and adjustments for confounding factors. Differences in eating habits and cooking methods, including duration and temperature, may also contribute to differences in findings. The traditional Korean diet, rich in carbohydrates and low in fat, featuring mixed grain rice, soup, kimchi, fish, and vegetables, may play a role in colon cancer prevention (41, 42). Further research is needed to clarify the association between traditional Korean food and colorectal adenoma.

Fish intake assessment in this current study was limited to fresh or unprocessed fish only. Processed fish consumption in the current study population was relatively low, and we observed a null association with colorectal adenoma; ORs (95% CIs) comparing top tertile vs. low tertile were 0.90 (0.67–1.21) for low-risk adenoma and 0.86 (0.52–1.42) for high-risk adenoma. Also, it would be informative to analyze the association between smoked meat and colorectal adenoma, as this information was not available in the current study. Future studies on this phenomenon should consider foodstuff and processing subtypes in assessing the association between diet and health outcomes. In addition, future dietary assessment instruments, including FFQ, can include a variety of fishes, taking into account food processing methods to clarify the actual association of subtypes of processing methods of foods in health and disease outcomes.

This study’s strengths include robust adjustments for confounding factors, and asymptomatic adults completed the FFQ just before screening colonoscopy, minimizing recall bias. The comprehensive colonoscopy, conducted by expert endoscopists and pathologists, improved polyp and adenoma detection and classification, reducing outcome misclassification. However, limitations exist. The cross-sectional design precludes establishing causation between total meat or fish intake and colorectal adenoma. Generalizability may be limited due to recruitment from a single hospital. Potential measurement errors in dietary assessments, reliance on baseline information, and the possibility of residual confounding factors, despite adjustments, are also acknowledged. Also, our findings should be interpreted with caution in light of shifting food exposure and changes in the magnitude of food and dietary consumption, given that the dataset for the current study is about a decade old. However, our primary findings relating to the associations of fish and meat consumption with colorectal adenoma are unlikely to be different given our crucial interest in the test of associations, not trends in consumption or disease patterns. Replicating our study findings in other Asian populations would be necessary.

Our findings extend the boundaries of knowledge on the potential implication of fish and meat consumption as primary sources of animal protein in the pathophysiology of colorectal cancer manifestation and progression, which is vital to guide clinicians and researchers for context-specific approaches targeted at unraveling the overall significance of dietary exposure in colorectal cancer prevention and control. Also, our study lends viable dietary information for supporting interventions in making a case for nutritional advisories and counseling for population-level improvement of healthy dietary and lifestyle practices for the primary prevention of colorectal cancer. Future longitudinal studies should consider histomorphological and molecular variations of colorectal adenoma in the colon and rectum to clarify the true association of fish and meat consumption in colorectal cancer outcomes.

In conclusion, this study demonstrated a significant association between higher fish intake and a reduced likelihood of high-risk colorectal adenomas, but no clear relationship was observed for red, processed and chicken meat with colorectal adenoma. Further research with a larger sample size and adherence to rigorous criteria is warranted to validate and expand upon these findings.

## Data Availability

The original contributions presented in the study are included in the article/Supplementary material, further inquiries can be directed to the corresponding author.
