# Supplementary material for: Fish and meat intake in relation to colorectal adenoma in asymptomatic Korean adults
Source: Front Nutr. 2024 Sep 4;11:1432647. doi: 10.3389/fnut.2024.1432647 (PMC11409847; doi:10.3389/fnut.2024.1432647)
Supplement: Supplementary file 1 [file Table_1.pdf]

Table S1: Characteristics of respondents by adenoma status in men and women

	Males		*Females*	
	Non-case group (n=627)	Adenoma group (n=388)	Non-case group (n=495)	Adenoma group (n=148)
Age (year), mean±SD	49.9±8.6	54.2±8.6	49.1±8.7	53.9±8.6
Education				
Middle school or less	12 (2.0)	10 (2.7)	25 (5.4)	14 (10.2)
High school	64 (10.5)	44 (11.8)	85 (18.2)	37 (27.0)
University Education & Postgraduate	532 (87.5)	320 (85.5)	356 (76.4)	86 (62.8)
Regular cigarette smoking, n (%)				
Never	156 (24.9)	96 (24.7)	454 (91.7)	136 (91.9)
Past	277 (44.2)	177 (45.6)	25 (5.1)	4 (2.7)
Current	194 (30.9)	115 (29.6)	16 (3.2)	8 (5.4)
Regular alcohol consumption				
Never	67 (10.8)	38 (9.9)	238 (49.5)	79 (55.6)
Past	42 (6.8)	30 (7.8)	37 (7.7)	7 (4.9)
Current	510 (82.4)	317 (82.3)	206 (42.8)	56 (39.4)
Alcohol intake (gram/day)	40.3±44.1	43.7±46.7	9.2±13.8	15.7±31.0
Physical Activity (METS mins/week)	1302.2±2406.9	1403.7±2457.0	1117.9±2557.6	1204.8±2718.8
Body mass index (kg/m^2), mean±SD	24.3±2.6	24.8±2.5	21.8±2.9	22.9±3.1
Waist circumference (cm), mean±SD	87.2±6.9	88.5±7.0	79.6±7.9	82.6±8.1
Systolic blood pressure (mmHg), mean±SD	117.9±12.6	119.5±12.8	111.1±13.0	116.2±13.2
Diastolic blood pressure (mmHg), mean±SD	77.5±9.8	78.8±9.6	70.5±9.7	72.5±10.6
High blood pressure, n (%)	266 (42.6)	188 (49.1)	97 (19.7)	50 (34.0)
Fasting plasma glucose (mg/dL), mean±SD	96.8±15.1	101.0±20.3	88.6±12.2	93.3±17.2
Glycated haemoglobin (%)	5.7±0.5	5.9±0.7	5.6±0.3	5.8±0.5
Diabetes, n (%)	195 (31.3)	154 (40.4)	60 (12.2)	29 (20.1)
Total Cholesterol (mg/dL), mean±SD	200.7±33.8	201.1±35.1	198.6±35.2	206.3±35.0
Triglyceride (mg/dL), mean±SD	117.6±71.6	124.2±84.8	72.2±37.0	83.3±47.1
HDL-cholesterol (mg/dL), mean±SD	49.1±10.2	48.9±9.6	57.7±10.7	55.6±12.2
LDL-cholesterol (mg/dL), mean±SD	129.1±30.1	127.9±29.4	120.4±31.0	129.7±30.7
Metabolic syndrome, n (%)	137 (21.9)	107 (27.9)	46 (9.3)	33 (22.3)
Colorectal cancer family history, n (%)	32 (16.3)	23 (24.7)	29 (19.9)	14 (26.9)
Total energy intake (kcal/day), mean±SD	1994.0±535.8	1941.6±541.6	1704.2±489.9	1719.3±525.0

SD, standard deviation; HDL, high-density lipoprotein-cholesterol; LDL, low-density lipoprotein-cholesterol; MET: the metabolic equivalent of task. High blood pressure was defined as systolic blood pressure ≥ 130 mmHg or diastolic blood pressure ≥85 mmHg or the use of antihypertensive drug treatment or a history of hypertension. Diabetes was defined as fasting blood glucose ≥ 100mg/dl or the use of glucose-lowering medications. Metabolic syndrome was defined as at least three of any of the following conditions; elevated waist circumference (≥90 cm – males ≥85 cm - females), elevated triglycerides (≥150 mg/dl or drug treatment for elevated triglycerides), reduced HDL-c (<40 mg/dL - males; <50 mg/dl - females), and high blood pressure or diabetes.

Table S2: Polytomous logistic regression models for the association of fish intake with odds of adenoma after additional adjustment for red meat

Quartiles of total fish intakes (g/day per 1000kcal)	Low risk adenoma group OR (95%CI)	High risk adenoma group OR (95%CI)	Low riak adenoma group OR (95%CI)	High risk adenoma group OR (95%CI)	Low riak adenoma group OR (95%CI)	High risk adenoma group OR (95%CI)	*P for interaction*
	All respondents		Males		Females		
Q1	1.00	1.00	1.00	1.00	1.00	1.00	0.16
Q2	1.09 (0.77, 1.54)	0.73 (0.42, 1.26)	1.03 (0.66, 1.60)	0.53 (0.28, 0.99)	1.17 (0.66, 2.10)	0.99 (0.26, 3.68)	
Q3	1.23 (0.87, 1.75)	1.15 (0.68, 1.94)	1.20 (0.76, 1.88)	0.80 (0.43, 1.48)	1.11 (0.62, 1.99)	1.84 (0.60, 6.68)	
Q4	0.95 (0.67, 1.36)	0.47 (0.25, 0.86)	0.98 (0.61, 1.57)	0.33 (0.16, 0.67)	0.80 (0.44, 1.45)	0.73 (0.20, 2.65)	
P-trend	0.63	0.03	0.76	0.003	0.38	0.75	
			Smoking Status				
			Never smoker		Ever Smoker		
Q1			1.00	1.00	1.00	1.00	0.66
Q2			1.17 (0.72, 1.92)	0.50 (0.18, 1.35)	1.06 (0.65, 1.74)	0.73 (0.36, 1.47)	
Q3			1.11 (0.68, 1.83)	1.16 (0.51, 2.65)	1.36 (0.82, 2.23)	0.94 (0.47, 1.90)	
Q4			0.86 (0.52, 1.42)	0.41 (0.15, 1.12)	1.07 (0.64, 1.81)	0.42 (0.19, 0.93)	
P-trend			0.38	0.15	0.83	0.04	
			Alcohol use Status				
			Non-drinker		Current drinker		
Q1			1.00	1.00	1.00	1.00	0.27
Q2			1.33 (0.73, 2.43)	0.59 (0.17, 2.07)	1.06 (0.69, 1.64)	0.75 (0.40, 1.42)	
Q3			1.17 (0.63, 2.16)	1.76 (0.64, 4.80)	1.32 (0.86, 2.04)	0.94 (0.50, 1.77)	
Q4			0.67 (0.35, 1.26)	0.42 (0.12, 1.46)	1.23 (0.79, 1.93)	0.52 (0.25, 1.07)	
P-trend			0.09	0.24	0.33	0.10	

OR, odds ratio; CI, confidence interval; Q, quartile. Models were additionally adjusted for energy-adjusted red meat intakes (g/day per 1000kcal, continuous) in addition to covariates in respective Model 1.

Table S3: Logistic regression models for the association of meat and fish intake with odds of all adenoma

	OR (95%CI)[1]		
Food (g/day per 1000kcal)	All respondents	Males only	Females only
Meat and fish			
Q1	1.00	1.00	1.00
Q2	1.03 (0.76, 1.40)	1.15 (0.79, 1.67)	0.89 (0.52, 1.51)
Q3	0.95 (0.70, 1.30)	1.11 (0.76, 1.62)	0.65 (0.38, 1.14)
Q4	1.06 (0.77, 1.45)	1.28 (0.87, 1.87)	0.67 (0.38, 1.19)
P-trend	0.82	0.26	0.11
Meats only			
Q1	1.00	1.00	1.00
Q2	1.06 (0.78, 1.44)	1.10 (0.76, 1.16)	0.85 (0.51, 1.45)
Q3	0.91 (0.66, 1.25)	1.13 (0.77, 1.65)	0.62 (0.35, 1.10)
Q4	1.15 (0.84, 1.58)	1.42 (0.96, 2.09)	0.68 (0.38, 1.20)
P-trend	0.47	0.08	0.15
Red meat only			
Q1	1.00	1.00	1.00
Q2	1.06 (0.78, 1.43)	1.05 (0.72, 1.54)	0.83 (0.49, 1.42)
Q3	0.95 (0.69, 1.30)	1.19 (0.81, 1.74)	0.59 (0.33, 1.05)
Q4	1.17 (0.85, 1.61)	1.35 (0.92, 1.99)	0.80 (0.46, 1.40)
P-trend	0.37	0.10	0.38
Poultry meat only			
Q1	1.00	1.00	1.00
Q2	1.43 (1.06, 1.93)	1.52 (1.05, 2.21)	1.47 (0.87, 2.48)
Q3	0.88 (0.64, 1.21)	0.90 (0.61, 1.32)	0.91 (0.52, 1.60)
Q4	0.95 (0.69, 1.30)	1.07 (0.72, 1.57)	0.83 (0.45, 1.51)
P-trend	0.22	0.62	0.26
Processed meat only			
T1	1.00	1.00	1.00
T2	1.06 (0.80, 1.40)	1.02 (0.70, 1.49)	0.99 (0.56, 1.72)
T3	1.14 (0.85, 1.52)	1.24 (0.90, 1.71)	0.85 (0.49, 1.46)
P-trend	0.40	0.18	0.54
Fish only			
Q1	1.00	1.00	1.00
Q2	1.01 (0.74, 1.39)	0.93 (0.64, 1.36)	1.61 (0.91, 2.84)
Q3	1.25 (0.92, 1.71)	1.10 (0.75, 1.61)	1.26 (0.71, 2.22)
Q4	0.85 (0.62, 1.18)	0.80 (0.54, 1.18)	0.95 (0.52, 1.73)
P-trend	0.29	0.27	0.36

OR, odds ratio; CI, confidence interval; Q, quartile. [1]Model was adjusted for age (in years, continuous), education (middle school or less, high school, university graduate and postgraduate), smoking status (never, past, current), alcohol intake (g/day, continuous), physical activity (METS mins/week, continuous), body mass index (kg/m^2, continuous), metabolic syndrome (no, yes), colorectal cancer family history (no, yes), total energy intake (kcal/day, continuous), and total fruit and vegetable intake (g/day per 1000kcal, quartiles)

Table S4: Logistic regression models for the association of fish intake with odds of all adenoma after additional adjustment for red meat

Quartiles of total fish intakes (g/day per 1000kcal)	OR (95%CI)[1]		
	All respondents	Males only	Females only
Q1	1.00	1.00	1.00
Q2	0.99 (0.72, 1.36)	0.88 (0.60, 1.29)	1.61 (0.91, 2.85)
Q3	1.21 (0.88, 1.66)	1.01 (0.68, 1.49)	1.26 (0.71, 2.24)
Q4	0.82 (0.59, 1.13)	0.72 (0.48, 1.08)	0.95 (0.52, 1.75)
P-trend	0.19	0.12	0.38

OR, odds ratio; CI, confidence interval; Q, quartile. Models were additionally adjusted for energy-adjusted red meat intakes (g/day per 1000kcal, continuous) in addition to covariates in respective Model 1.

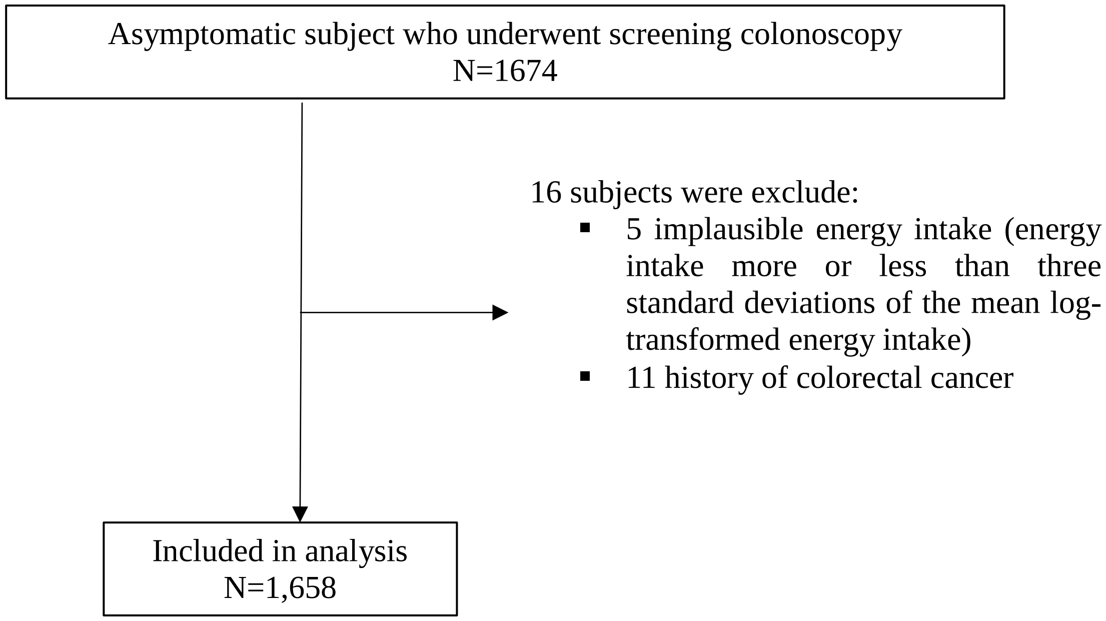

Figure S1. Flowchart describing Participant selection in the study
